# The point of no return in the Emotional Stop-Signal Task: A matter of affect or method?

**DOI:** 10.1371/journal.pone.0315082

**Published:** 2024-12-05

**Authors:** Ambra Coccaro, Antonio Maffei, Killian Kleffner, Patrick Lennon Carolan, Antonino Vallesi, Giulia D’Adamo, Mario Liotti

**Affiliations:** 1 Padova Neuroscience Center, University of Padova, Padova, Italy; 2 Department of Developmental and Social Psychology, University of Padova, Padova, Italy; 3 Institute of Cognitive Science, University of Colorado Boulder, Boulder, CO, United States of America; 4 Department of Psychology, Simon Fraser University, Burnaby, Canada; 5 Department of Psychology, Saint Mary’s University, Nova Scotia, Canada; 6 Department of Neuroscience, University of Padova, Padova, Italy; 7 Department of Neuroscience, University of Parma, Parma, Italy; Universita degli Studi di Trento, ITALY

## Abstract

An affective variant of the Stop-Signal task was used to study the interaction between emotion and response inhibition (RI) in healthy young participants. The task involved the covert presentation of emotional faces as go stimuli, as well as a manipulation of motivation and affect by inducing a negative mood through the assignment of unfair punishment. In the literature on emotion and RI, there are contrasting findings reflecting the variability in the method used to calculate the RI latency, namely the Stop-Signal Reaction Time (SSRT). In fact, previous studies found both facilitatory and detrimental effects of affective manipulations over RI. However, they did not use the most robust SSRT estimation approach, namely the integration, casting some doubts on the reliability of the inferences. For these reasons, the present research draws attention on how the effect of the emotional manipulation may be due to a biased SSRT estimation. Specifically, the focus of our study was on how the effect of emotion on the SSRT may vary according to different estimation procedures, the mean and two variants of the integration method. We predicted that the effect of the emotional manipulation in the SST would depend on the SSRT estimation method employed. Indeed, a significant effect of emotion was only found when SSRT was estimated with the mean method. We conclude that the mean method should be avoided in the study of emotion and RI because it overestimates SSRT. Rather, the integration approach should be used for future research in this field, while also factoring in information about the participants’ strategy in emotional contexts that require greater effortful control and offer a challenge to self-regulation both in health and disease.

## Introduction

Executive Functions (EFs) constitute a set of top-down high-order cognitive processes that enable goal-directed behavior. According to leading models, core executive functions include inhibitory control, set-shifting, and working memory [1–3]. Inhibitory control encompasses two sub-components: Interference control (selective attention and cognitive inhibition) and response inhibition (RI; suppressing a dominant response due to a change in goals [4–6]). In turn, response inhibition includes two sub-domains, i.e., reactive inhibition, the ability to react to a signal to stop, and proactive inhibition, the ability to modulate reactive inhibitory control in advance according to current context and one’s goals [7, 8].

In recent years there has also been a growing interest in how emotions and affect may impact and modulate inhibitory control. The focus of the present study was to contribute to the understanding of the mechanisms by which emotion and motivation affect the ability to react to a signal to stop, that is, reactive response inhibition in a young adult healthy sample. Different versions of an “emotional” stop-signal task have been employed to investigate emotional inhibitory control in healthy and clinical populations ([9–24] see Table 1). Such studies yielded largely variable findings, returning a very complex picture. While the majority of them reported impaired inhibitory control as a result of emotional manipulations [14–21], some investigations found improved performance in emotional contexts compared to neutral ones [9–13]. Therefore, it seems that an emotional manipulation could lead to either a RI facilitation or can have a detrimental effect on it. In addition, there is also limited evidence of no effect at all of emotion on RI [22, 23].

**Table 1:** Scientific literature on SST and emotion.

Authors	Stimulus	Emotion relevance	Method SSRT	Effect of emotion	DOI
**Allen et al. 2021 [24]**	IAPS scenes (neg, pos)	Overt	Median	Detrimental	10.1177/2398212821105826
**Camfield et al. 2018 [23]**	IAPS scenes (neg, pos, neutral)	Covert	Not specified	No effect	10.1016/j.ijpsycho.2017.12.008
**Ding et al. 2020 [21]**	faces (sad, neutral)	Overt	Median	Detrimental	10.3389/fnbeh.2020.00119
**Herbert et al. 2011 [16]**	words (neg, pos, neutral)	Overt	Not specified	Detrimental	10.4236/jbbs.2011.13020
**Kalanthroff et al. 2013 [19]**	IAPS scenes (neg, neutral)	Covert	Mean	Detrimental	10.3389/fnhum.2013.00078
**Krypotos et al. 2011 [17]**	IAPS scenes (neg, neutral)	Covert	Integration	Detrimental	10.3389/fpsyg.2011.00278
**Littman et al. 2017 [12]**	IAPS scenes (neg, neutral)	Covert	Mean	Facilitatory	10.1371/journal.pone.0186774
**Padmala at al. 2010 [15]**	state-dep motivation	None	Median	Detrimental	10.1016/j.neuropsychologia.2009.10.017
**Patterson et al. 2016 [20]**	IAPS scenes (neg, neutral)	Covert	Median	Detrimental	10.1007/s00221-016-4709-2
**Pawliczek et al. 2013 [10]**	faces (angry, neutral)	Covert	Median	Facilitatory	10.1016/j.neuroimage.2013.04.104
**Pessoa et al. 2012 [9]**	state-dep motivation	None	Median	Facilitatory	10.1037/a0024109
**Rebetez et al. 2015 [18]**	faces (joy, angry, neutral)	Covert	Integration	Detrimental	10.1080/02699931.2014.922054
**Sagaspe et al. 2011 [22]**	faces (fear, neutral)	Covert	Median	No effect	10.1016/j.neuroimage.2011.01.027
**Senderecka et al. 2018 [11]**	sounds (fear, neutral)	Covert	Integration	Facilitatory	10.3758/s13415-017-0546-4
**Verbruggen et al. 2007 [14]**	IAPS scenes (pos, neg, neutral)	Covert	Mean	Detrimental	10.1080/02699930600625081
**Williams et al. 2020 [13]**	faces (fear, hap, neutral)	Covert Overt	Median	Facilitatory	10.1080/02699931.20201793303

A potential explanation of this striking inconsistency in past research could lie in the methodological variability, especially with regards to how RI is measured. The above-mentioned studies measured RI through the hallmark Stop-Signal task (SST), in which serially presented stimuli are evaluated by the participants who have to respond or withhold the answer on a trial-by-trial basis. The time required to inhibit the ongoing action, called Stop-Signal Reaction Time (SSRT), is an index of RI latency and efficiency, according to the “independent horse race model” [5]. This model entails that RI can be conceptualized as a race between two independent processes: the go and the stop “horses”. The outcome of the inhibitory process depends on the relative completion time of the two competing processes. The time window between the presentation of the go and the stop stimuli, namely the stop signal delay (SSD), is varied in order to collect an individual estimate of SSRTs. The contrasting findings regarding the interactions between emotions and RI mirror the variability in the methods employed in order to estimate the SSRT. The independent race model offers two main nonparametric approaches to calculate SSRT: the mean and the integration methods [25]. The former calculates the index by subtracting the mean SSD during stop trials from mean Response Times (RTs) on go trials. The latter approach, instead, integrates the distribution of all RTs during go trials and the probability of responding to the stop signal. A third approach emerging from the literature, called the median method, involves the subtraction of the mean SSD from the median of the go RTs [26].

Verbruggen et al. [25] suggested that the two main methods have a mathematical justification based on the race model, whilst the median approach has not been proven to be as stable as the others, unless the inhibition function (the probability of responding on stop trials as a function of the stop-signal delay) is symmetrical. Furthermore, the authors have shown how SSRT is influenced by the distribution of go RTs, and suggested a strategy to obtain the best estimation of the process called RI. In fact, when the go RT distribution is skewed, the mean method will overestimate the SSRT. Although the integration method was found to be less influenced by the right-skewed distribution of go RTs, it does seem to be affected by strategic slowing. A possible solution involves the use of a variant of the integration method that tries to take into account these characteristics and it will be presented in the method section. These analytic considerations should be particularly important in the context of emotions, where a skewness in the distribution of go RTs and a general strategic slowing is expected, highlighting the importance of using the most appropriate method to calculate SSRTs. For these reasons, we conducted a qualitative review of the literature on research employing the emotional SST to assess the impact of emotion on RI. We found a total of 16 studies, as shown in Table 1. Half of the studies (8 out of 16) employed the median method for estimating the SSRT; Three studies adopted the integration method, the first including all go RTs, the second including only RT during valid go trials, while the third study did not specify the ranking procedure for go RTs. The mean method was employed in three research articles, while the remaining two studies did not specify the estimation process. Considering the great variability in the estimation methods of the SSRT, we decided to compare different calculations in order to substantiate the hypothesized effect of emotions over the EF.

Please also note from Table 1 that further variability in the results from existing studies derives from the use of different emotional manipulations: The majority of studies employed images of emotional scenes from the International Affective Picture System (IAPS: [12, 14, 17, 19, 20, 23, 24]), but some other studies utilized emotional faces [10, 18, 21, 22], emotional words [16] or emotional auditory sounds [11]. Few studies addressed state-dependent, or induced changes in affect or motivation [9, 15, 20]. Perhaps more importantly, in some studies the emotional dimension of the Go stimulus was task-relevant, with participants responding to the emotional or valence content of the Go stimulus [16, 21, 24]. In the majority of studies, however, emotion was task-irrelevant, with participants responding to another simultaneous or immediately consecutive dimension of the Go target stimulus [10, 12, 14, 17–19, 22]. The processing strategy may be particularly critical, since recent studies using the emotional Go-NoGo task suggest that a key factor for obtaining a reproducible effect of emotional stimuli is task relevance as a key determinant of motor readiness or inhibition [7, 27]. Similarly, overt face emotion categorization tasks more consistently yield behavioral effects of emotion relative to studies involving covertly processed face expressions [28].

The paradigm employed in the present research was a variant of the Emotional Stop-Signal Task (EMOSS) involving the presentation of human facial expressions overlayed with either yellow or pink transparent masks (go stimuli). The participants were instructed to respond to the color of the mask while ignoring the face; in other words, the emotional content was task-irrelevant. In 30% of the trials, after the presentation of the go stimulus, a Stop-Signal (SS) occurred, and participants were required to inhibit the response to the preceding go stimulus. As in previous studies [10, 18, 21, 22, 24] we explored the stimulus-driven effects of face expressions with different emotional valence: negative (angry), positive (happy) and neutral (no emotion).

Furthermore, the EMOSS involved a second emotional manipulation to achieve a state-dependent change of affect and motivation level, i.e., a negative emotion induction [9, 26, 29], since emotion can direct executive control also through state-dependent changes in affect/motivation [9, 26, 29]. To fully characterize and maximize the impact of emotion on RI, we opted to incorporate both methods. This approach is the same as previously employed in an emotional Go/No-Go task, proven to be a robust manipulation of the participant’s emotional state [26, 29]. In fair blocks, participants gained/lost equal points for correct/incorrect responses. In contrast, in the unfair block, devised to induce negative emotion, they received the same amount for a correct answer, but an incorrect response led to losing all points. The temporary loss of all points triggered negative emotions, as confirmed by self-report scales and changes in brain electrophysiology [26, 29].

### Aims and hypotheses

The general aim of the present study was to contribute to the knowledge of the mechanisms involved in affective reactive inhibitory control in a SST. We adopted a variant of the emotional SST, which incorporated a state-dependent manipulation of affect and motivation, by contrasting a negative affect condition (unfair block) to a neutral baseline affect condition (fair blocks, [26, 29]). In order to best characterize the interaction between reactive response inhibition and emotion, the focus was on how such emotional manipulation would influence different methods to estimate the latency of response inhibition in the SST. To test the hypothesized effect on estimation approaches, three different methods (mean, median and integration) were used to calculate the latency of the inhibitory mechanism in emotional and non-emotional conditions.

We predicted that, since the negative emotion manipulation would require a new strategy to optimally perform the SST, it would primarily impact go processes. Although the model underpinning the SSRT assumes independence between go and stop processes, the resulting SSRT estimate, measuring the inhibition latency, could be biased by the cognitive strategy employed by the participants in go trials, as proven by the simulations performed by Verbruggen et al. [25]. To address this problem, in the present investigation we computed the SSRT with three different methods: 1) the mean method, which does not account for potential changes in the performance in go trials, 2) the integration method, which controls for omissions during the go trials, and 3) the revised version of the integration method proposed by Verbuggen et al. [25], which also takes into account progressive (strategic) slowing during the go trials. We hypothesized that the frustrating block would influence the go performance as indexed by the increased proportion of omission errors and longer go RTs. The proportion of successful stop trials would increase in the emotional block because of the hypothesized strategy adopted by participants during go trials.

Furthermore, to test the influence of the presentation of distracting emotional expressions in the SST, we predicted that emotional stimuli would differentially affect performance during go and stop processes. Neutral images should result in shorter RT compared to happy and angry faces. Indeed, emotional stimuli attract attention that is diverted away from the task, as proven by the “interference effect” reported in previous emotional SSTs, similar to what is typically reported in other emotional cognitive control tasks [16, 25]. This manipulation should also impact the stop performance in an opposite fashion.

We also predicted that the SSRT, depending on the estimation method, would give rise to contrasting results, with an overestimation of the mean method in the emotional block, compared to the two versions of the integration approach. Indeed, if omissions increased while there was a concomitant slowing of go RTs during the affective block, the mean method would become unstable, and not as valid as the integration approach due to the influence of go RT distribution on this estimation method (i.e., mean). Thus, it could possibly signal that emotion did not directly influence stop performance but instead the go process, that in turn spuriously impacted the estimation of the latency of the inhibitory mechanism.

## Methods

The experimental procedure was approved by the ethics committee of the School of Psychology of the University of Padova (protocol n° 3446), including a written consent prior to collecting the data. Data collection started on the 18^**th**^ of April 2020 and ended on the 12^**th**^ of July 2021. The datasets generated and analyzed during the current study are available at **https://github.com/AmbraC/DOC/blob/026a2844cbbd469e7eb0af5865a44617331e4e73/dataExtended.csv**.

### Participants

Participants [N = 67; age range: 19–30 years old; mean age = 24.84 years (SD = 3.56), male/female ratio = 21/46] were recruited through social media targeting undergraduate and graduate students willing to participate in behavioral experiments. Participants reported normal or corrected-to-normal vision, and they admitted to no history of psychiatric or psychological conditions. An a-priori power analysis estimated a sample size of 50 to achieve a power of 80% to reach an effect size of η^2^_p_ = .17, based on the literature [16]. The sample size was increased to 67 since previous research indicated that about 10% of participants can violate the independence assumption, which may constitute an issue in the SST, that will be explained later. The assumption postulates that RTs during go trials must be longer than RT during unsuccessful stop trials [5].

### Measures

**Fig 1:**
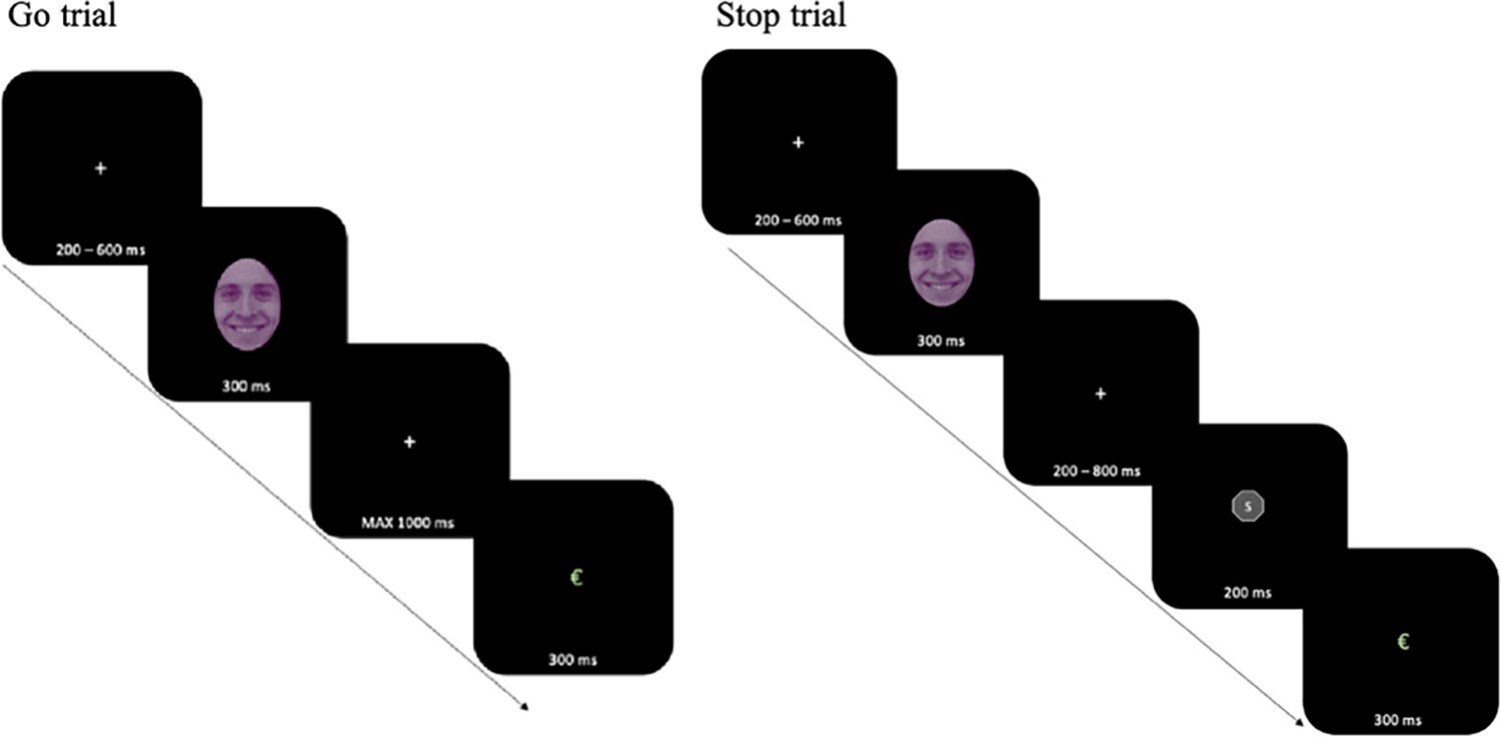
Experimental procedure.

Participants signed a written consent form and completed a self-report demographic survey. Afterwards, they performed a 30-minute online task. The EMOSS task variant was developed using E-prime 2.0.8.22 (Psychological Software Tools, Pittsburgh, PA) and adapted to an updated version of the software (3.0.1.13). Before starting the actual experiment, a 3-minute practice session was performed (40 trials: 12 stop and 28 go trials) to ensure that participants understood the task. During EMOSS, the primary task (Go stimuli) involved faces with various expressions with a superimposed color mask, and participants were instructed to perform a two-choice color discrimination while ignoring the emotional content of the face (covert emotional processing). A secondary task involved the inhibition of the ongoing response to the primary one (in 30% of the trials). The go stimuli were a series of faces extracted from the Karolinska Directed Emotional Facial (KDEF) database [30], as depicted in Fig 1. It contained 10 faces each for both sexes with happy, angry, and neutral expressions, for a total of 60 images that were used. Each face was gray-scaled with superimposed oval frames covering jaw and hairlines, and for each of the 60 images there were two versions based on the colored mask imposed over the face, that is, pink or yellow. Ultimately, 120 different stimuli were presented to participants.

On each trial, one of the images was shown at the center of the screen: stimulus duration was 300 ms and participants responded with the keys ‘z’ and ‘m’ of the computer keyboard. Order of the response keys was counterbalanced across participants. Responses were accepted up to 1000 ms after stimulus onset. In 30% of cases, after the presentation of a face, a SS in the form of a gray hexagon with a white ‘S’ in the middle, appeared at central fixation for 200 ms, indicating that participants should withhold their response to the preceding face stimulus. Each trial ended with central feedback displayed for 1000 ms, indicating a wrong (“X”) or right answer (“€”). Participants received/lost points for right/wrong responses for either go or stop stimuli.

**Fig 2:**
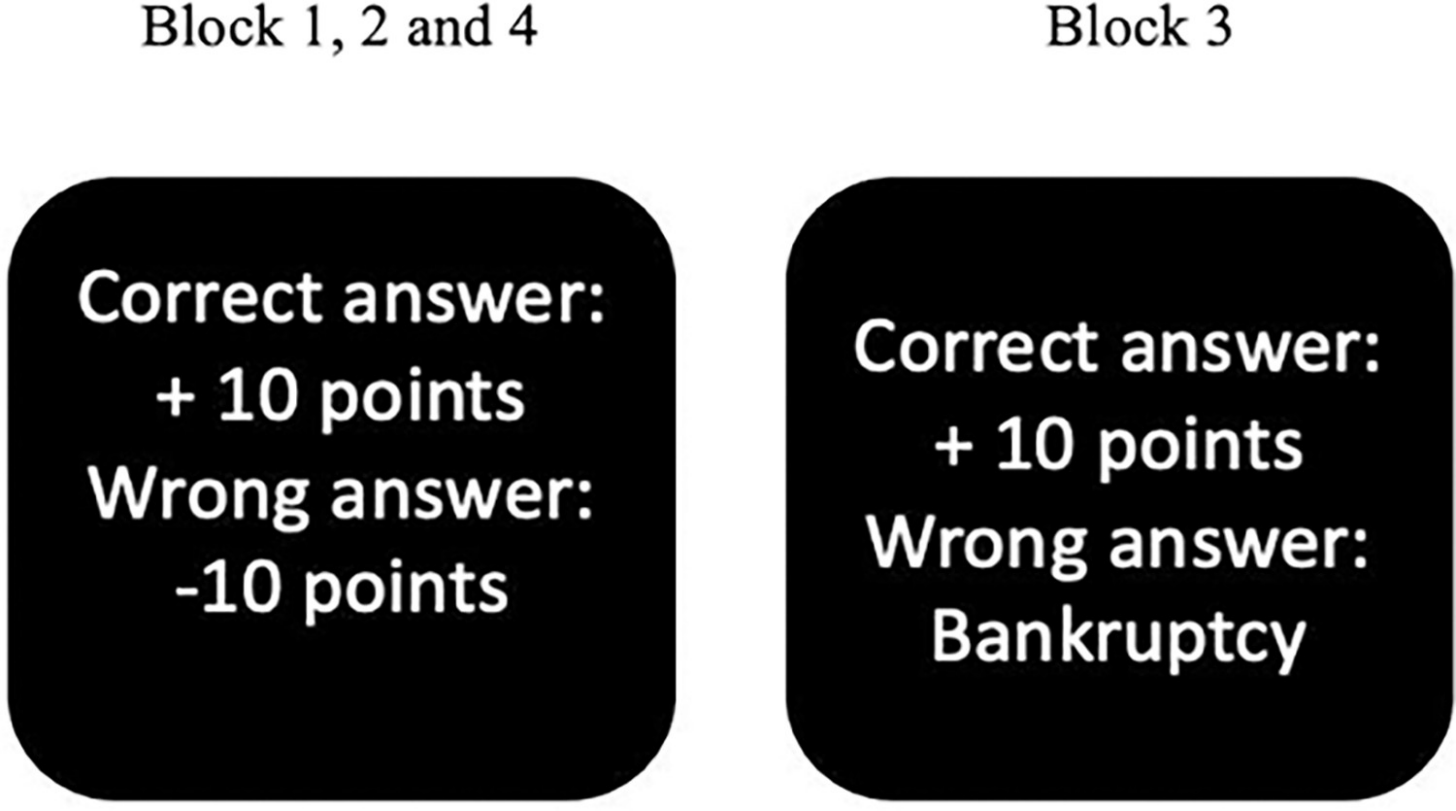
Block procedure: The assignment of points changes in block 3 (right) and the other blocks (left).

Throughout the task, a tracking procedure was implemented to adaptively adjust the stop signal delay (SSD) by 100 ms based on the performance of the participants enabling around 60% of accuracy in stopping the ongoing action. The SSD ranged from 200 to 800 ms, with a step of 100 ms and a starting latency of 200 ms. This strategy prevents participants from predicting and therefore waiting for the stop signal, while also allowing to obtain about 50% of successful inhibitions overall [21, 29]. The experiment included four blocks administered in fixed order as presented in Fig 2. Block 1, a baseline condition without emotional content, only presented faces with neutral expression. After that, blocks 2 to 4 included three emotional face expressions, i.e., positive (happy), negative (angry) and neutral. Importantly, blocks 1, 2 and 4 were fair blocks, where participants received or lost 10 points for correct or incorrect responses. In contrast, block 3 was unfair, in that 10 points were assigned for each correct answer, but a wrong response led to bankruptcy: all gained points were lost, in order to induce a state of high frustration in the participant [29, 31].

Each of the 4 blocks comprised 160 trials (120 go and 40 stop trials) presented pseudorandomly. Every block included 4 mini-blocks of 40 trials. After each mini-block there was a short pause, during which the total points gained was presented, and participants were reminded about the current rule relating to the assignment of points, and when a rule was about to change. After that, they were asked to rate their level of frustration and motivation on a 5-point Likert scale from 1 (“Not at all”) to 5 (“Extremely”).

### Techniques and procedures

Data collection was carried out online through an application developed by E-prime, namely e-go, which gives the ability to control for time delay in the presentation of stimuli and in the collection of the response: Participants were excluded from the analysis if they exceeded a given threshold. No such a case was found. Participants accessed the platform Jotform.com which contained consent form, instructions, and the software to perform the task. The EMOSS lasted 30 minutes and the total time for participants to complete the study was 45 minutes.

### Data processing and analysis

Data were excluded from the analysis if at least one of the following conditions was met:

- There was a violation of the independent assumption [5]: mean RT during go trials had to be longer than mean RT during unsuccessful stop trials;
- The probability of inhibiting when the SS was present was lower than .35;
- The percentage of successful go trials was lower than 75%.

These characteristics found in performance could have been representative of the misunderstanding of the instructions or of the application of strategies that will impact the estimation of SSRT in the SST. One strategy refers to waiting for the SS and therefore violating the independent assumption and/or having low accuracy rate during go trials. Or the alternative strategy is to prioritize the performance during go trials and thus obtain insufficient accuracy rate for stop trials.

A total of seven participants were excluded: five because of the violation of the independent assumption, and two due to low accuracy rate in go trials. SSRT was calculated following three different methods, two of which were the classical mean and the integration approaches. The formula of the former is the following:

$$SSRT\;mean=\mu_{go}-\mu_{SSD}$$ (1)

Where *μ_go_* is the mean RTs during Go trials and *μ_SSD_* is mean Stop-Signal Delay.

The integration method, instead, uses the following equation:

$$SSRT\;integration={nthRT}_{go}-\mu_{SSD}$$ (2)

Where *nthRT_go_* is number of RT in the RT distribution of go trials multiplied by the probability to respond to the signal result in nth and *μ_SSD_* is mean Stop-Signal Delay.

In the integration approach, the total number of go trials is multiplied by the probability to respond to the signal: the resulting number indicates the position of the rank-ordered go RTs (the nth RT) in the distribution. The mean SSD is subtracted from the nth RT to obtain the SSRT. The method currently most valuable, as supported by the simulations by Verbruggen et al. [25], is the integration method with the replacement of omissions with maximum RTs, and the equation is the one just presented, with the exception that in considering the go RT distribution, the omissions are replaced with the maximum RT. There is, also, a third estimation approach that is the block-wise integration method which estimates the SSRT for each mini-block (40 trials) and then average for the whole block (160 trials) in order to reduce the effect of strategic slowing.

Two methods were used to calculate the probability of stopping an ongoing action: the first embodies the canonical approach by which the number of successful stop trials is divided by the total number of stop trials. Nevertheless, the measure does not consider other intervening factors, such as the omission errors during go trials, which consequently increase the probability of performing a later successful inhibition. Therefore, the second method involved an adjustment of the index based on the number of omissions made by the participant. The estimation was computed as follows:

$$p(respond|signal)=\frac{1-(p(inh|sig)-p(omis|go)}{p(omis|go)}$$ (3)

The equation is calculation the probability to respond to a stop signal where *p*(*inh|sig*) is representing the probability to respond during stop trial and *p*(*omis|go*) is the probability to omit the response during go trials.

To further substantiate the hypothesized strategic slowing during go trials, we also included the post-error slowing, that is, the difference between mean RTs after correct stop trials and mean RTs after unsuccessful stop trials. Post-error slowing in the stop-signal task refers to the tendency for participants to slow their responses on trials after making an error. When an error occurs (i.e., failing to inhibit the response when needed), participants often slow down on the next trials. This slowdown is thought to be a proactive strategy to enhance accuracy and prevent further mistakes by increasing caution and adjusting their approach. The mechanisms behind post-error slowing include increased caution, enhanced error monitoring, cognitive reappraisal, and higher cognitive load, all of which contribute to more deliberate and careful responses.

In the present research, a linear mixed effects model was applied because, unlike repeated-measure ANOVA, it accommodates missing data while controlling for the dependency structure due to repeated measures design [32]. The independent variables taken into account were: SSRT (mean and the two integration methods), proportion of unsuccessful stop and its variant corrected for omissions, RTs and accuracy during go trials, post-error slowing, proportion of omission errors. The within-subject factors comprised reward-based Block (4 levels) and emotional Images (3 levels), with the latter being excluded in the estimation of the SSRT because the number of trials for each condition would have produced an unstable estimation. A random-intercept was fitted to model repeated measurements over the subjects. Significance of main effects was assessed with an F test, using the Satterthwaite approximation for degrees of freedom. Post-hoc t-tests were applied to all significant results found in the preceding step, and they were corrected for multiple comparison with the False Discovery Rate. A correlational analysis was carried out in order to verify the relationship between RT and omissions during go trials. Finally, we tested if there was a significant difference between the mean level of motivation and frustration in the different blocks, expecting to find in block 3 the higher level of frustration and the lower level of motivation. In addition, this approach allowed us to see if the recovery block (4^th^) still displayed the same motivation/frustration pattern of block 3 or if the participants actually recovered from it.

## Results

### Mood induction checks

#### Frustration ratings

**Fig 3:**
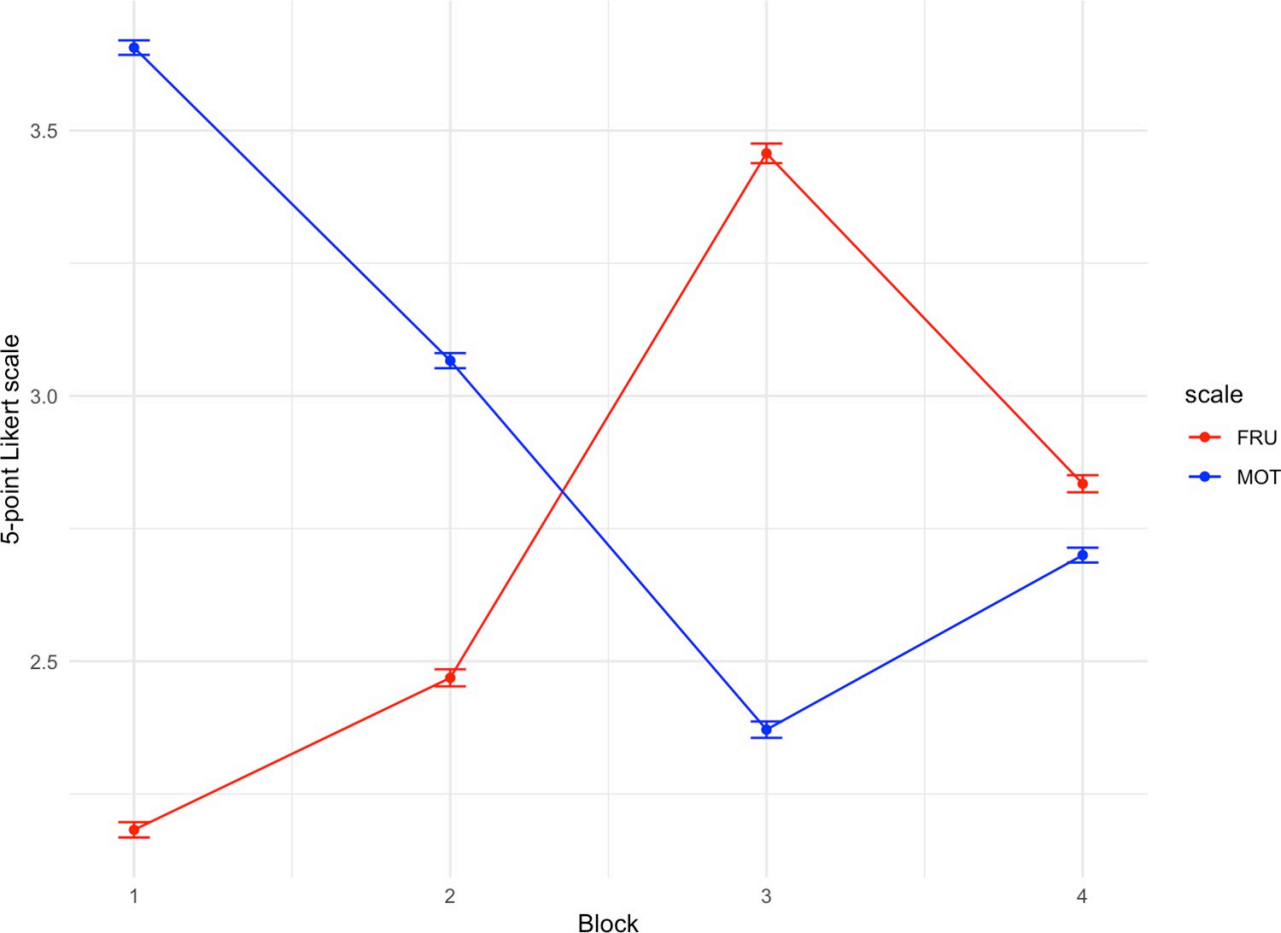
Levels of frustration (in red) and motivation (in blue) among blocks.

A significant effect of block (F(3,177) = 36.34, p < .001, 95% CI [.04, 1.00], ***η*^2^** = .08) was found in self-reported frustration. Post-hoc tests highlighted significant differences (p < .001) between the first two blocks (1 and 2) and the last two blocks (3 and 4), with no difference between blocks 1 (M = 2.16, SD = .96) and 2 (M = 2.46, SD = 1.08) and between blocks 3 (M = 3.46, SD = 1.24) and 4 (M = 2.84, SD = 1.07). Results are shown in Fig 3.

#### Motivation ratings

A main effect of the block (F(3,177) = 40.97, p < .001, 95% CI [.08, 1.00], ***η*^2^** = .14) was also found for self-reported motivation ratings. A post-hoc analysis revealed an almost linear significant decrease of motivation level from block 1 (M = 3.65, SD = .92) and 2 (M = 3.06, SD = .96) to block 3 (p < .01), with no significant changes between block 3 (M = 2.37, SD = 1.03) and block 4 (M = 2.69, SD = .94). Results are shown in Fig 3.

Combining the information from the two scales, participants experienced more frustration and less motivation in block 3, and during the recovery block 4 negative affect with greater frustration and low motivation were still lingering in spite of the switch to a fair condition.

Firstly, Table 2 displays the summary of the measures employed in the present research.

**Table 2:** Summary of behavioral performance.

Block	Go RTs	Stop RTs	stop-signal delays	Go accuracy	Stop accuracy
**1**	928.22	786.20	353.81	0.93	0.60
**2**	947.30	829.91	384.51	0.93	0.60
**3**	964.57	834.77	382.55	0.90	0.63
**4**	955.84	834.36	392.53	0.91	0.60

### Behavioral results

Please note that the stimulus-driven emotional effect due to the presentation of the emotional faces on the estimation of the SSRT could not be reliably assessed because of insufficient number of trials in each category. In fact, Verbruggen et al. [25] concluded that a minimum of 25 stop trials are necessary for a stable estimate of the SSRT. Therefore, reported here are the behavioral results concerning the state-dependent frustration manipulation, independent of emotional valence of the Go stimuli.

#### Go trials

**Fig 4:**
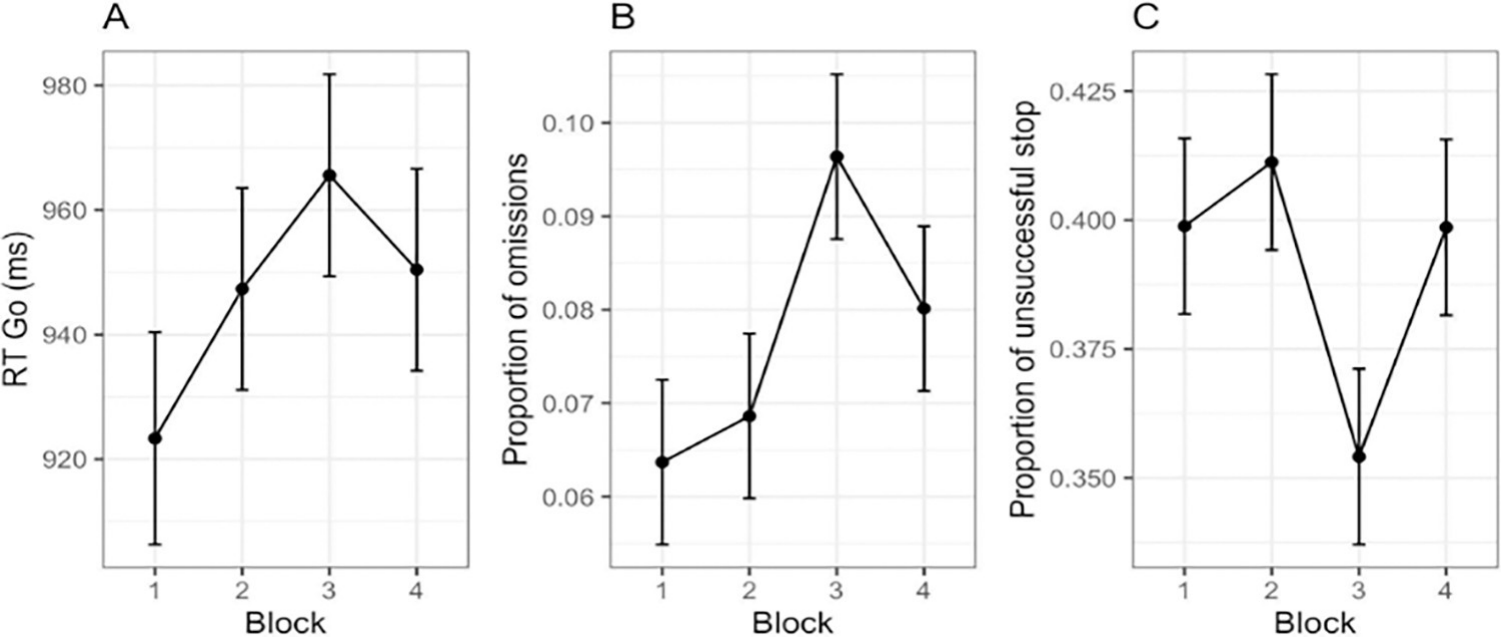
Mean Response Time (panel A) and proportion of omission errors (panel B) during go trials. Panel C presents the averaged proportion of unsuccessful stop trials.

A significant main effect of block (F(2,472) = 4.90, p < .01, 95% CI [.03, 1.00], *η*^2^ = .05) on RTs was found during go trials as shown in Fig 4, panel A. Post-hoc t-tests highlighted a significant difference between block 3 (M = 964, SD = 133) and all the other levels: block 1 (p < .001; M = 928, SD = 140), block 2 (p < .001; M = 947, SD = 149) and block 4 (p < .05; M = 956, SD = 134). No differences were instead found in RTs to the different emotional expressions presented (F(2,528) = 0.00, p = .9, 95% CI [.00, 1.00], *η*^2^ = .00), nor for the block x image interaction (F(4,528) = 0.57, p = .5, 95% CI [.00, 1.00], *η*^2^ = .00).

There was a main effect of block (F(3,177) = 4.17, p < .01, 95% CI [.01, 1.00], *η*^2^ = .06) in the proportion of omission errors (Fig 4, panel B). Post-hoc t-tests revealed a significant difference between block 3 (M = .096, SD = .11) and all the others (block 1: p < .05, M = .063, SD = .04; block 2: p < .05, M = .069, SD = .04) except for block 4 (p = .4, M = .080, SD = .06). Fig 4 summarizes three main findings (See S1 Fig in the supporting information for distributions), related to each other: RTs and omissions during go trials, and the proportion of unsuccessful inhibitions that will be presented in the next paragraph.

**Fig 5:**
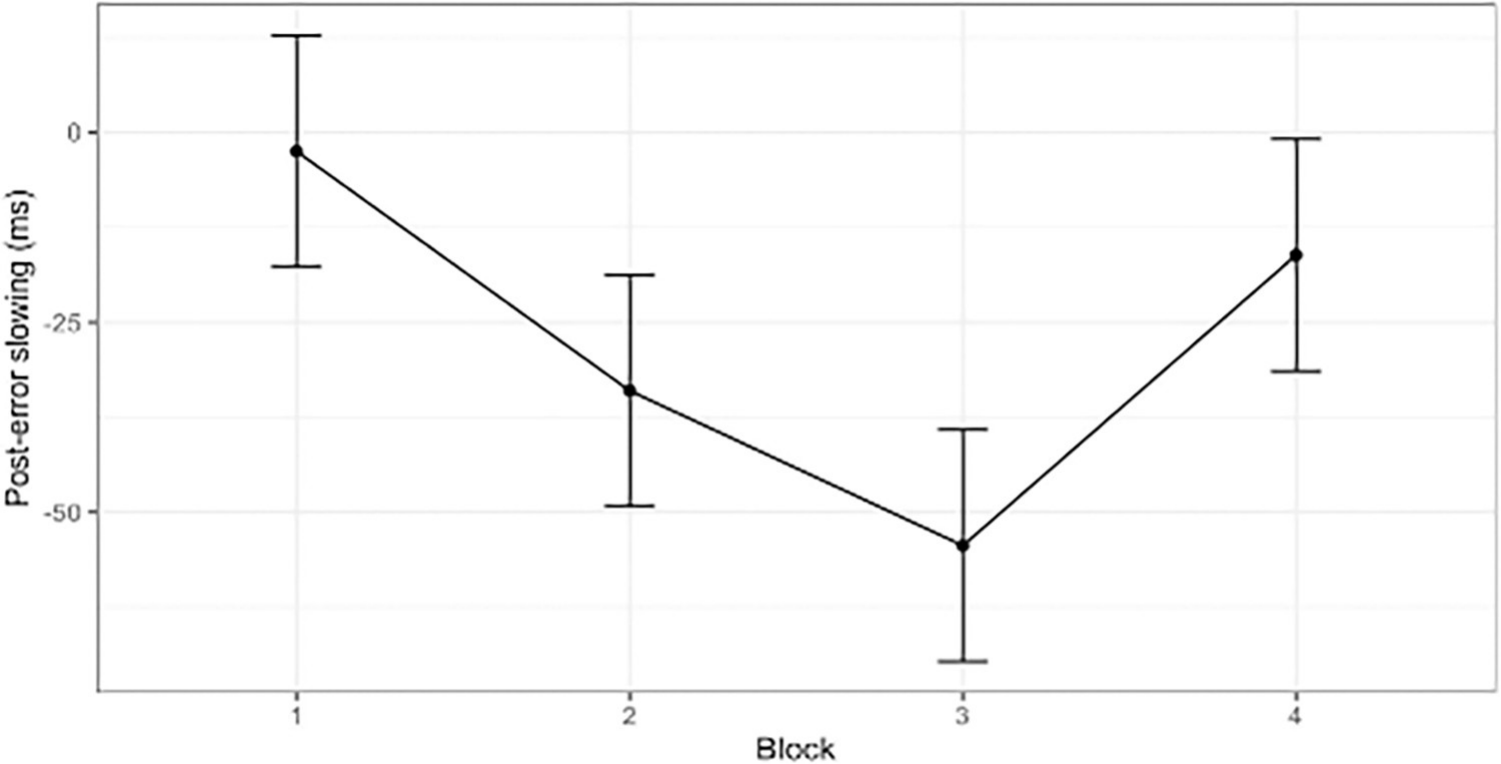
Post-error slowing among blocks.

Finally, the analysis on post-error slowing revealed an effect of the block that only approached significance (F(3,177) = 2.42, p = .06, 95% CI [.00, 1.00], *η*^2^ = .04). Fig 5 shows a trend for greatest post-error slowing in block 3 (See S2 Fig in the supporting information for distributions).

#### Stop trials

**Fig 6:**
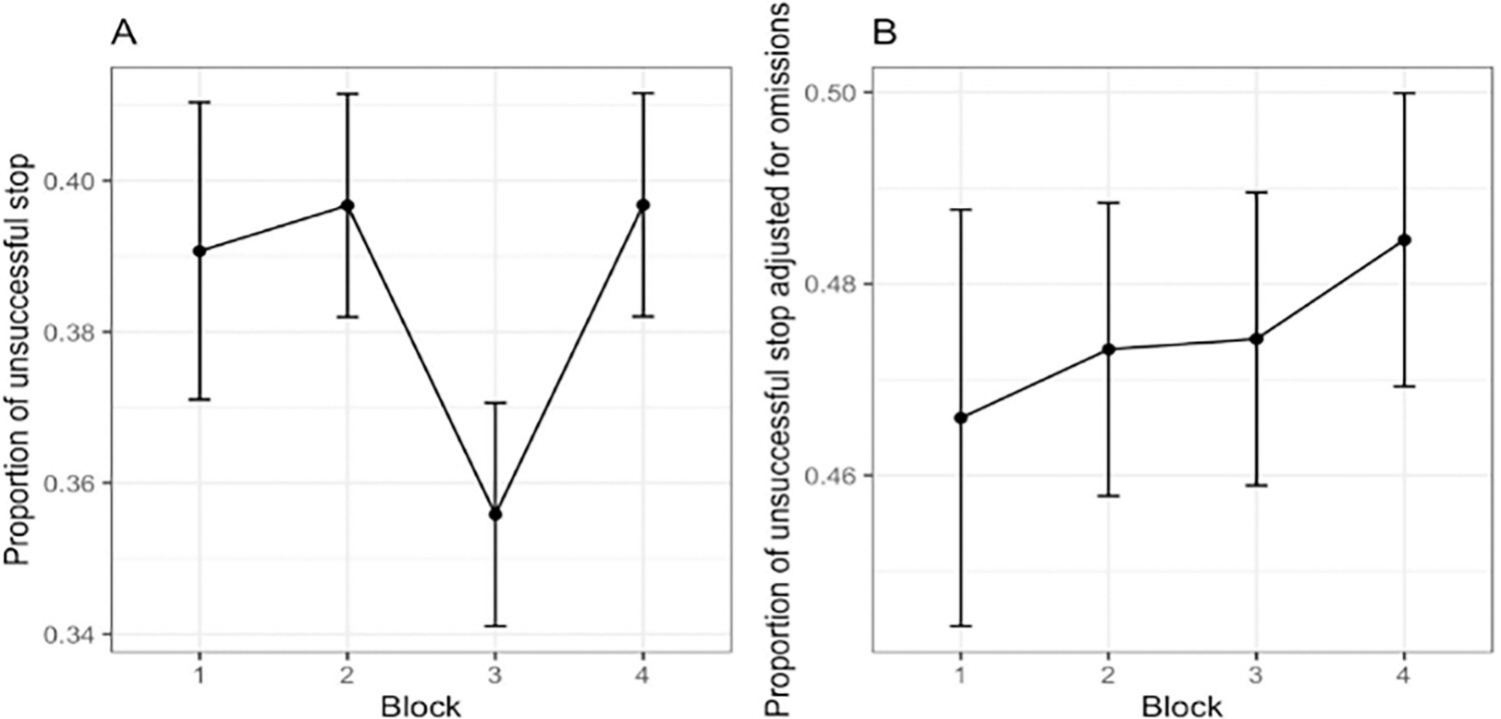
Proportion of unsuccessful stop trials (panel A) and proportion of unsuccessful stop trials corrected for omission errors (panel B).

The probability of responding to the SS significantly varied among blocks (F(2,504) = 9.72, p < .001) as depicted in Fig 6, panel A. Post-hoc t-tests (p < .001) confirmed a significant difference between block 3 (M = .36, SD = .12) and both blocks 2 (M = .40, SD = .14) and 4 (M = .40, SD = .14), but no difference was found with block 1 (p = .7, M = .39, SD = .15). However, when we considered the same index adjusted for omission errors, no significant effects were found (F(2,504) = 0.31, p = .8, 95% CI [.01, 1.00], *η*^2^ = .06).

In fact, Fig 6 highlights how the calculation of the proportion of unsuccessful stop trials is influenced by omission errors.

**Fig 7:**
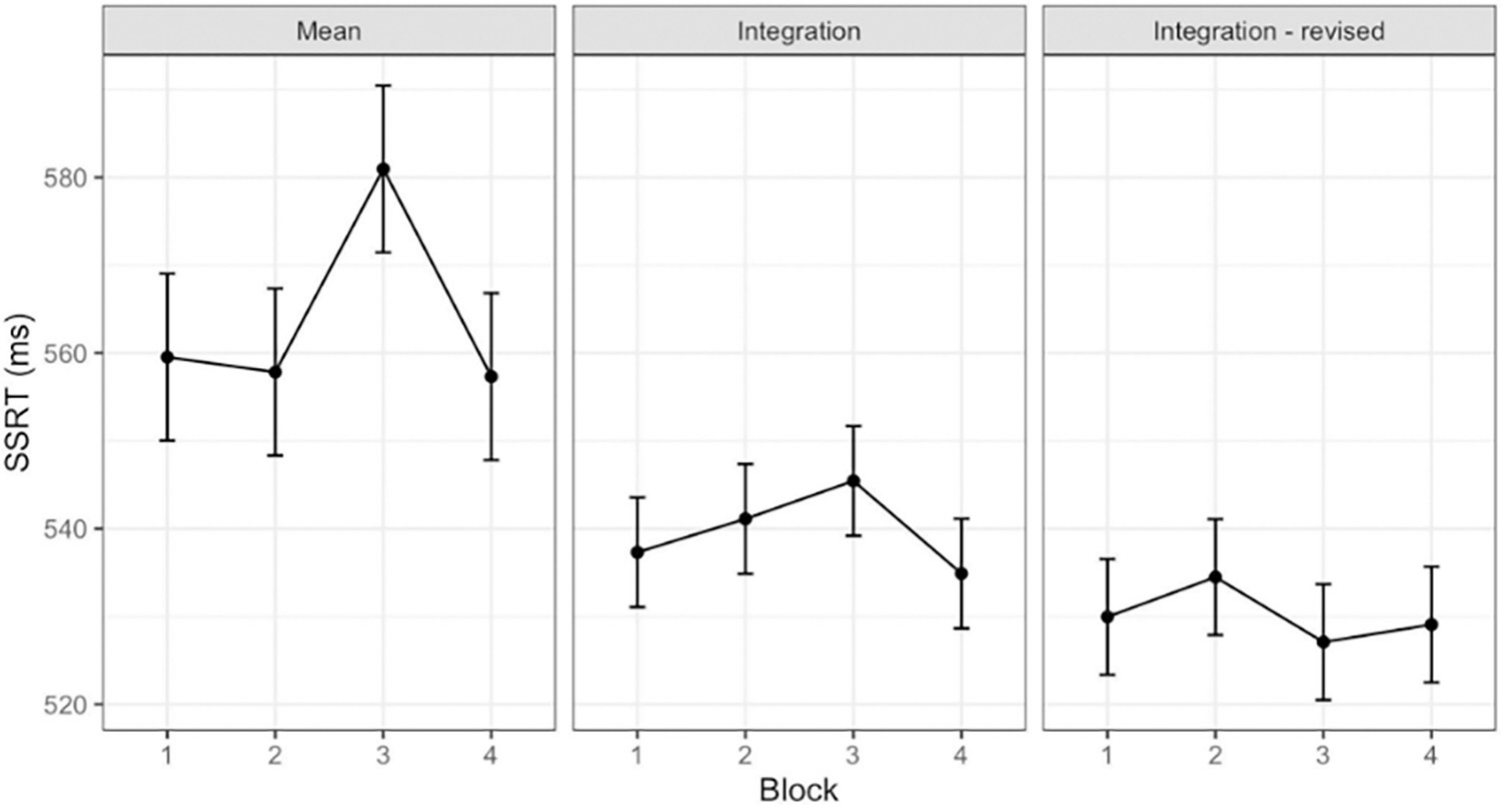
Stop-Signal Reaction Time calculated with mean, integration and block-wise integration methods.

The mean calculation returned a significantly longer SSRT (F(3,177) = 2.73, p < .05, 95% CI [.00, 1.00], *η*^2^ = .05) during block 3, whilst the integration (F(3,177) = 0.85, p = .3, 95% CI [.00, 1.00], *η*^2^ = .00) and the averaged SSRT (F(3,177) = 0.17, p = .7, 95% CI [.00, 1.00], *η*^2^ = .00) yielded no effect at all of the frustrating block, as shown in Fig 7 (See S3 Fig in the supporting information for distributions). This could be one of the causes of the contrasting results found in the literature.

## Discussion

The present study explored the mechanisms involved in affective reactive inhibitory control in a SST that manipulated the emotional context in order to produce an unfair, frustrating condition or a fair, non-emotional condition in healthy adults. In particular, it focused on how negative affect would influence three different methods used to calculate the latency of the inhibitory mechanism in emotional and non-emotional conditions.

The results of our investigation highlighted that, while the mean method estimated longer SSRTs in the emotional context, the two variants of the integration approach yielded no effect at all. On the other hand, go RTs were sensitive to the induction of a negative mood exerted during the frustrating block and specifically they were the slowest compared to the rest of the task. A possible explanation of the present effect is that the mood induction block influencing the motivation to accurately complete the task resulted in a global slowing of the performance.

As reported in the introduction, Inhibitory Control as indexed by the SST includes two subtypes of inhibition, namely proactive RI and reactive RI [33, 34]. Proactive RI entails a strategy to increase the rate of success in stop trials, yielding longer RTs also during go trials in order to increase the rate of success in stop trials, whilst the reactive strategy refers to the flexible adjustment of behavior to balance between the two competing goals. We interpret the results on RTs in the unfair blocks as a consequence of changing the RI strategy from a reactive to a proactive modality in order to beat the stop signal. In support of this interpretation, we found an increased proportion of omission errors during the frustrating block which may be the result of exceeding slowing. Such proactive strategy enacted during the emotional block in turn is reflected in an overestimation of the latency of RI measured by the mean method. Indeed, the calculation of the SSRT is intrinsically related to both go and stop mechanisms. Consequently, the mean method produced a significantly longer stop latency, as a result of the slowing strategy during go trials, that was not found in the two versions of integration SSRTs.

By adjusting the performance during go trials, with longer RTs and more omission errors, participants obtained a higher probability of inhibiting the response in the frustrating block. Nonetheless, it was not the case when this probability was adjusted for go omissions. Furthermore, there is a proven effect of the variability and the distribution of go RTs over the estimation of SSRT with the mean method. When there is a global slowing during go trials which in turn causes a temporal shifting of the successive mechanism, that is, response inhibition, the mean method does not account for it and overestimates the inhibitory latency. This phenomenon could partially explain the great variability found in the literature on the interactions between emotions and RI in healthy populations.

The foregoing arguments summarize why the current investigation draws attention to the potential bias underlying the “fictitious inhibitory differences” [35] between emotional and non-emotional contexts: depending on the choice of the estimation approach, a researcher can describe longer SSRT in the emotional condition or no effect of it. Considering that there is a growing awareness about a bias toward the publication of positive results [36, 37], this phenomenon could have potentially influenced the choice of the method to estimate the latency of RI. In addition, due to the simplicity of application of the mean and median methods, most of the research in the field has used them in order to study how emotion impacts RI. We therefore suggest that when a SST is used in emotional conditions, it is advised to use the integration method in order to correctly account for the impact of an emotional manipulation on RI.

Hence, a main limit of past research stems from the estimation method of the latency of RI that calls into doubt the validity of the inferences made on the effect of the emotional manipulations. Indeed, the median method seems to be the most utilized in the research line pertaining to the interactions between emotions and RI, even considering that it is not mathematically justified nor is it explained why it should be a valid method in the context of the horse-race model. In fact, the model makes predictions only regarding mean RTs and mean of the inhibition function. The median method is thus mathematically justified only in case of symmetrical inhibition function. If there is an asymmetry in the inhibition function, for instance because the go RT distribution is skewed, the median will underestimate the mean of the inhibition function while overestimating the SSRT [35].

Consequently, the mean and the median method should be abandoned in the estimation procedure for the SSRT due to the excessive influence of the shape of go RT distribution that is expected to be modulated by affective contexts. In fact, the interactions between emotions and RI could be the cause of the slowing strategy and omissions that in turn produce the skewness of the go RT distribution.

Verbruggen, Chambers and Logan [35] in the context of the non-emotional SST, demonstrated that differences in the skewness of go RT distributions between groups or conditions could lead to spurious inhibitory effects. As clearly described by Verbruggen et al. [25] for the classic non-emotional SST, the integration method with the replacement of omissions, in conjunction with the adjustment procedure, is generally more reliable and less biased by the right tail of the go RT distribution, in comparison to the mean approach. In past research applying the integration method, it appeared that the authors have ranked only the go RTs of correct answers, ignoring omissions, and other types of errors. These mistakes are important indices in the study of emotions because they signal an adjustment in performance as a consequence of the emotional manipulation.

Liddle et al. [33] used a SST designed to investigate the effect of different motivational levels over performance, and they found that in the high motivational condition participants adjusted the performance through a global slowing, even if it was a penalized option in terms of reward they could earn, because this allowed responses to be calibrated so as to fall within a time-window that maximizes the probability of success, regardless of trial type. This result highlights the importance of considering such a strategy as a naturally occurring phenomenon that cannot be discouraged, as proposed by Verbruggen [25] with the aim of limiting the skewness of the go RT distribution and therefore the bias over the estimation of SSRT. The consensus guide on SST [25], published in 2019 by experts on the task, has established the basis for a more cautious and responsible approach to calculate SSRT. Nonetheless this guide should be integrated with the literature pertaining to affect and motivation, since the factors influencing the estimation methods are the same phenomena that we expect to find when emotions are at play. Our work contributes to the integration of the important information contained in the consensus guide [25] with the literature on emotions in order to stimulate a shift in methodology also in a specific research field that uses an emotional manipulation similar to ours.

Our results may have important implications for the study of the relationship between emotional RI and underlying clinical dimensions useful in psychopathology. Traditionally, impulsivity, as probed by the stop-signal task, has been linked to externalizing psychopathology, such as in Attention Deficit Hyperactivity Disorder (ADHD [38]). Recent research [39] highlights how participants with high scores of hyperactivity and inattention in fact display increased RT variability, rather than a slower processing speed, when carrying out a SST. This finding is in contrast with the literature reporting longer SSRTs in ADHD [40, 41], opening new possibilities in approaching the study of ADHD as well as other clinical conditions to further investigate the specificity of the index. Furthermore, a recent body of literature has linked impulsive reactivity also to emotion, and therefore to internalizing aspects of psychopathology, proposing a broader vulnerability role towards various forms of psychopathology [42]. Recent theoretical models propose the construct of negative urgency as a tendency to respond impulsively to negative affect, with high levels of negative emotionality and disinhibition, implicated in a broad range of psychopathological disorders [24]. Indeed, self-report measures of Negative Urgency have been found to correlate with poor negative emotional response inhibition in an Emotional Stop Signal task [24]. In future works, it would be important to ascertain how the methodological biases discussed in the present study may impact or inform the existing theoretical models of emotion-cognition interactions in psychopathology, possibly improving them [24, 42].

## Limitation and future directions

Using the E-Prime platform for online data collection can be challenging due to compatibility issues with various browsers and operating systems, which may impact the consistency of the data collected. Nonetheless, we asked participants to always use the same browser and we were able to check that the delay of stimuli presentation due to differences in the hardware was under 100 ms and, most importantly, that it was constant throughout the task. Nonetheless, the lack of controlled lab conditions could affect participant engagement and adherence to experimental protocols, potentially compromising data validity and reliability.

The heterogeneity of the design of the SST makes the comparison with the existing literature challenging, mainly due to different emotional manipulations and staircase procedures. For these reasons, the generalizability of our results should be considered in regard to similar SSTs that use irrelevant emotional manipulation.

A second limitation of this study is its focus on a young adult healthy population. Future research should apply the EMOSS to healthy or clinical cohorts with externalizing and internalizing symptoms, and also use longitudinal designs, to explore its validity and potential applicability to diverse clinical settings.

Third, we cannot entirely exclude that the RI performance decrement following the mood manipulation may also be due to other cognitive/attentional processes at play in the demanding frustration blocks (which varied only for the meaning of the punishment feedback). A future study may want to replicate the present findings using a mood induction technique that precedes and does not alter at all the SST [43, 44].

Finally, further studies may also employ in the EMOSS a task-relevant version in order to possibly increase the stimulus-driven impact of the emotional faces, since there is evidence for Go-No Go tasks that only task-relevant fearful expressions may increase the RTs and rate of commission errors relative to happy faces [7, 27, 28].

## Conclusion

The present research pointed out that the study of the interaction between emotion and response inhibition is biased by the estimation method used to calculate the latency of the inhibitory process. By comparing different estimation metrics, we conclude that the emotional impact on response inhibition is mainly due to the estimation approach rather than the emotional manipulation per se. We further discuss the more suitable method in the research line exploring the relationship between an affective manipulation and motor inhibitory control mechanisms.

Whilst a form of standardization in the estimation procedure for SSRT is expected in the near future, in the meanwhile we urge for the employment of an estimation approach to the SSRT that is suitable for the aim of the study. Finally, the proactive strategy found only in the emotional condition should be studied as a relevant index in order to understand which characteristics of the environment elicit such behavior and how clinical/subclinical populations adjust their behavior in order to complete the task. In fact, there are studies reporting that individuals with high internalizing and externalizing symptoms show similar effects on go and stop processes that are comparable to the effects we found due to the emotional manipulation [26, 29].

## Supporting information

S1 Fig Distributions of Go RTs in ms, proportion of omissions and unsuccessful stop. (TIF)

S2 Fig Distributions of post-error slowing. (TIF)

S3 Fig Distributions of Stop-Signal Reaction Time calculated with mean, integration and block-wise integration methods. (TIF)
