# Supplementary figures and images for: The point of no return in the Emotional Stop-Signal Task: A matter of affect or method?
Source: PLoS One. 2024 Dec 5;19(12):e0315082. doi: 10.1371/journal.pone.0315082 (PMC11620632; doi:10.1371/journal.pone.0315082)

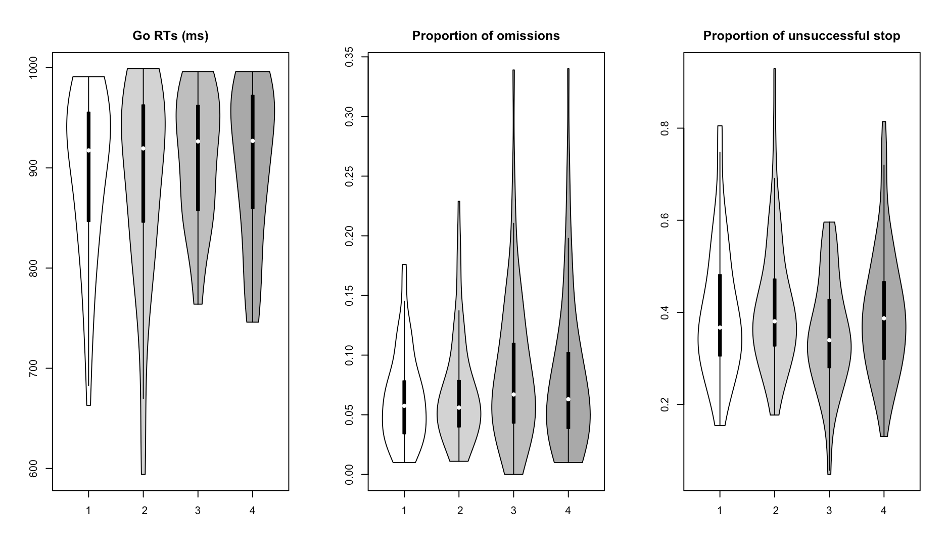

Supplement: S1 Fig — (TIF) [file pone.0315082.s001.tif]

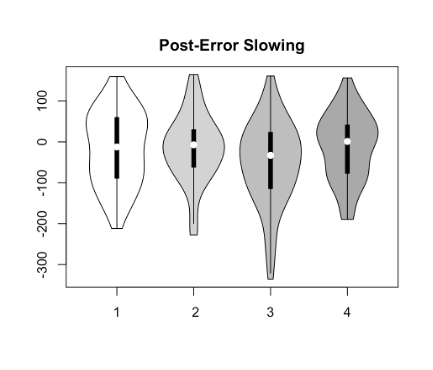

Supplement: S2 Fig — (TIF) [file pone.0315082.s002.tif]

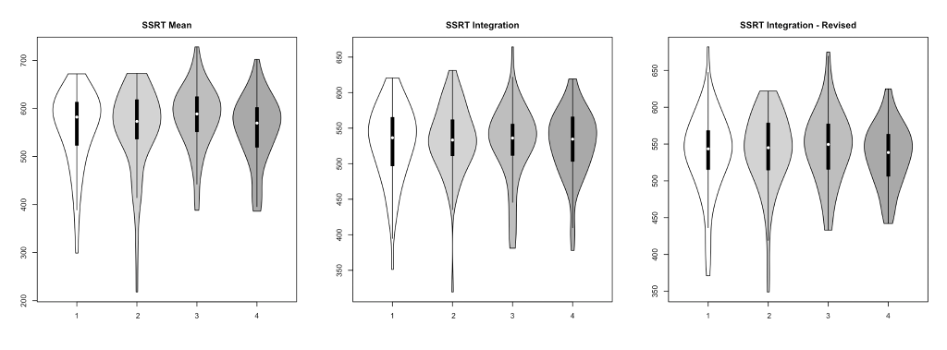

Supplement: S3 Fig — (TIF) [file pone.0315082.s003.tif]
